# Effects of the Pinggan Qianyang Recipe on MicroRNA Gene Expression in the Aortic Tissue of Spontaneously Hypertensive Rats

**DOI:** 10.1155/2015/154691

**Published:** 2015-08-25

**Authors:** Guangwei Zhong, Xia Fang, Dongsheng Wang, Qiong Chen, Tao Tang

**Affiliations:** ^1^Institute of Integrated Traditional Chinese and Western Medicine, Xiangya Hospital, Central South University, Changsha 410008, China; ^2^Department of Geriatrics, Xiangya Hospital, Central South University, Changsha 410008, China

## Abstract

The present study aimed to investigate the relationship between miRNAs and in spontaneously hypertensive rats (SHR) vascular remodeling and analyze the impact of the Pinggan Qianyang recipe (PQR) on miRNAs. Mammalian miRNA microarrays containing 509 miRNA genes were employed to analyze the differentially expressed miRNAs in the three groups. MiRNAs were considered to be up- or downregulated when the fluorescent intensity ratio between the two groups was over 4-fold. Validation of those miRNAs changed in SHR after PQR treatment was used by real-time quantitative RT-PCR (qRT-PCR). Compared with the normal group, a total of 32 miRNAs were differentially expressed by more than twofold; among these, 18 were upregulated and 14 were downregulated in the model group. Compared with the normal group, there were a number of 17 miRNAs which were significantly expressed by more than twofold in the different expressions of 32 miRNAs; among these, 10 were downregulated and 7 were upregulated in the PQR group. qRT-PCR verified that miR-20a, miR-145, miR-30, and miR-98 were significantly expressed in the three groups. These data show that PQR could exert its antihypertensive effect through deterioration of the vascular remodeling process. The mechanism might be associated with regulating differentially expressed miRNAs in aorta tissue.

## 1. Introduction

Hypertension, a lifelong condition, is one of the most common cardiovascular diseases. Among patients treated by the authors, the prevalence of hypertension in 15 to 69-year-old patients is 23.4%, greater than the current estimate of patients with hypertension in China [1]. Because hypertension is an important risk factor for coronary heart disease and stroke, damage to the vital organs such as the heart, brain, and kidneys can be avoided or minimized by preventing and controlling high blood pressure [2]. A Chinese medicine scholar has successfully explored the pathogenesis of spontaneous hypertension and various therapy approaches, including the Pinggan Qianyang recipe (PQR), a Chinese medicine recipe for calming the liver and suppressing yang [3]. PQR, which originated from the use of Tianma Guoteng beverages, has been used to treat essential hypertension with satisfactory results [4]. Recent research has found that Chinese herbal medicines that involve PQR have a beneficial effect on reducing blood pressure and recovering circadian rhythm in essential hypertension patients [5, 6]. However, the underlying mechanism of these therapeutic effects remains unknown.

miRNAs are a class of highly conserved, noncoding, small-molecule RNAs, consisting of about 22 nucleotides each. They adjust protein levels by promoting mRNA degradation or inhibiting mRNA translation. miRNAs thus participate in many important biological processes throughout the body [7, 8]. miRNAs are involved in cell proliferation, differentiation, migration, and apoptosis [9, 10]. Cordes et al. found that reducing miRNA-143 levels could inhibit adipocyte differentiation in vitro, suggesting that miRNAs may play a significant role in the renin-angiotensin system (RAAS)—an important modulator of systemic blood pressure [11]. Some miRNAs, including miR-1, miR-145, miR-122, miR-221, and miR-222, have been linked to vascular endothelial dysfunction [12]. Others have been linked to the regulation of vascular smooth muscle cells; these include miR-145, let-7d, miR-24, miR-26a, and miR-146 [13]. The miRNAs miR-1, miR-155, and miR-208 have significant effects on the RAAS [14]. Therefore, a new strategy for hypertension treatment might involve maintenance and restoration of stability by targeting corresponding miRNA expression in the organ of interest.

To elucidate the association between miRNA expression and PQR treatment for essential hypertension, we carried out analysis of miRNA gene expression in aortic tissue from SHR that had received PQR intervention. We tested the hypothesis that PQR plays an antihypertensive role by regulating miRNA expression in rat aortic tissue. This research may also provide new insights into potential therapeutic targets to prevent and treat hypertension.

## 2. Materials and Methods

### 2.1. Animals and Drugs

Forty 16-week-old male spontaneously hypertensive rats (SHR) and 20 male Wistar (WKY) rats (Vital River Laboratory Animal Technology Co., Ltd., Beijing, China) of the same age were housed in a sterile environment at a temperature of 21 ± 1°C and a relative humidity of 50% ± 10% in a 12-hour day-night cycle. Both groups of rats had been fed standard rat chow and water until they were 16 weeks old. All animal study protocols were approved by the Animal Care and Use Committee of Central South University (201303117) and followed the animal management rules set out by the Ministry of Health, China, and the US National Institutes of Health Guide for the Care and Use of Laboratory Animals. The PQR medication recipe was composed of Rhizoma Gastrodiae, Ramulus Uncariae cum Uncis, Concha Haliotidia, Concha Ostreae, and Radix Achyranthis Bidentatae; all components were purchased from the Department of Pharmacy, Xiangya Hospital, Central South University. One gram of extract was equal to 4.25 g of crude material.

### 2.2. Animal Groupings and Treatments

The WKY rats and SHR were arbitrarily separated into three groups: the normal group (*n* = 20), the model group (*n* = 20), and the PQR group (*n* = 20). Rats in the PQR group were administered PQR at a dose of 5.0 mg·kg^−1^·d^−1^ by gastrogavage. The others were given an equal volume of distilled water. For all groups, the administration course lasted 4 weeks. All animals were used for the miRNA analysis and verification study. Forty SHR were randomly divided into two groups and were given 5.0 mg/kg of PQR by gastrogavage once daily for 4 weeks; normal saline was given as the negative control.

### 2.3. Blood Pressure Detection

Systolic blood pressure (SBP) was measured in all rats as previously described [15]. Tail-cuff plethysmography (TCP) with a rat tail blood pressure monitor was used. The SBP of each rat was measured five times—once before treatment and 1, 2, 3, and 4 weeks after treatment. At every time point, the mean of the lowest three values within 5 mmHg was regarded as the SBP value.

### 2.4. Histological and Morphological Assay

Rats were anesthetized with 10% chloral hydrate (400 mg/kg, intraperitoneal injection) at the end of each week of whole-day drug administration. The thoracic aorta below the aortic arch of each rat was stripped and clipped. A portion was fixed in 8% neutral formaldehyde, embedded in paraffin, sectioned at 5 *μ*m, and stained with the hematoxylin-eosin (HE) and Masson methods [16]. Light microscopy was used to image each cross-sectional slice, of which there were five per rat. Each vascular ring in the perpendicular position and the vessel media wall were observed. The images were observed under a Leica imaging system (Leica Microsystems GmbH, Wetzlar, Germany). The media thickness (MT) and inner diameter (LD) were measured, and the ratio of media thickness to inner diameter (MT/LD) was calculated. Other parts of the thoracic aorta were removed from the adventitia and were promptly refrigerated at −80°C for miRNA assay.

### 2.5. RNA Microarray and Hybridization

*RNA Extraction*. Total RNA was extracted by a one-step method using TRIzol (Invitrogen, USA) following the manufacturer protocol, concentrated using isopropanol precipitation, and quantified using a spectrophotometer and agarose gel electrophoresis. The polyethylene glycol (PEG) method was used to isolate and purify 50 *μ*g of total RNA.

*Fluorescently Labeled miRNA*. miRCURY LNA array labeling kit (Exiqon, Denmark) was used. Total RNA (10 *μ*g) was added to 2 *μ*L of Hy_3_ fluorescent label solution and 2 *μ*L of labeling enzyme, mixed by pipetting, and then incubated at 65°C for 15 min to terminate the labeling process.

*miRNA Microarray Hybridization*. A miRCURY LNA array labeling kit using Macro Kit (ID # 208000V7.1) and hybrid box II (ID # 40080) was purchased from Exiqon. Biochip slides and cover slips were purchased from Ambion, Inc. (USA). miRNA microarray hybridization was performed according to the miRCURY LNA array kit instructions: 10 *μ*L of total RNA was added to 10 *μ*L of 2x hybridization buffer and incubated for 3–5 min at 95°C. Then, 20 *μ*L of the hybridization solution was placed on a microarray slide and completely covered with a Bioarray Lifter Slip coverslip. The microarray slide was placed into the Hybridization Chamber II in a horizontal orientation and bathed at 60°C for 16 h. Following incubation, hybridization samples were removed from the microarray slides with a wash solution. Each of 509 miRNAs was detected by three replicate probe spots on each microarray slide, for a total of six measurements per miRNA per sample after repeated fluorescence exchange.

*Image Acquisition and Quantification*. Each microarray (chip) was rinsed and immediately dried, then illuminated by a single 635 nm beam and scanned by a GenePix 4000B dual laser scanner (Molecular Devices, LLC, USA). Image files were saved in TIFF format. The data were analyzed by GenePix Pro 6.0 software (Molecular Devices, LLC, USA). After preprocessing, the data were normalized to the same interchip global mean. Finally, the differentially expressed genes were analyzed by SAM (Significance Analysis of Microarrays, version 2.1). We used the following screening conditions: false discovery rate of <5%, and expression differences of ≥2-fold.

### 2.6. Target Prediction Methods

Predicted miRNA target genes were determined by four software programs: miRanda (http://www.microrna.org/), miRBase Target Database (http://microrna.sanger.ac.uk), and Target Scan (http://www.targetscan.org/) [17]. Outputs varied among the programs. Genes predicted by at least two programs were selected as predicted miRNA target genes.

### 2.7. Quantitative RT-PCR

Differentially expressed miRNAs, selected according to ≥2-fold upregulation or downregulation by microarray analysis, were measured by qRT-PCR using RNA-tailing and primer extension. Briefly, 2 *μ*g of RNA was added to 2.5 U/*μ*L of poly (A) polymerase and 1 mmol/L of ATP and incubated in water for 30 min at 37°C. PCR primers were designed according to miRNA sequences indicated by the aforementioned online software programs (2.6). U6 small nuclear RNA in the rats was used as an internal control gene. Real-time PCR reactions were amplified in a 96-well PCR fluorescence analyzer (MJ real-time PCR instrument, Bio-Rad Laboratories, Inc., USA). Samples were predenatured for 5 min at 95°C, denatured for 20 s at 94°C, annealed for 20 s at 58°C, and extended for 30 s at 72°C, for a total of 40 cycles, with each sample analyzed in triplicate. The specific product in each PCR reaction was confirmed by the amplification curve. Quantification of relative gene expression was determined by the standard 2^−ΔΔCt^ method: relative gene expression = 2^−(ΔCtsample−ΔCtcontrol)^.

### 2.8. Statistical Analysis

All results are presented as the mean ± standard deviation. All experiments were repeated three times. An independent sample *t*-test was applied when only two groups were compared, whereas comparisons between more than two groups were made by analysis of variance (ANOVA) followed by a Bonferroni posttest. Differences were considered significant at the level of *P* < 0.05.

## 3. Results

### 3.1. PQR Significantly Decreased SBP

**Figure 1:**
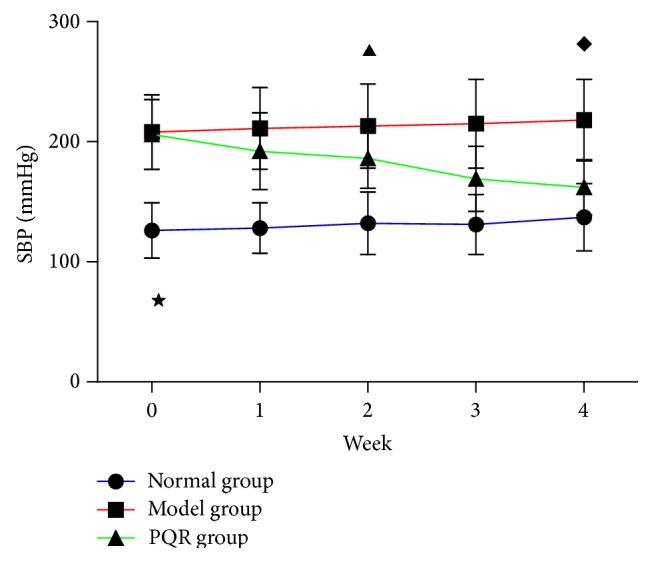
SBP changes in WKY rats or SHR receiving an i.a. of PQR or distilled water at various times. Data are shown as the mean ± SD for twenty rats of each group. *P* values for statistical significance were as ^★^ *P* < 0.01, compared with the model group; ^▲^ *P* < 0.05, and ^*◆*^ *P* < 0.01, compared with the PQR group, respectively.

At the beginning of treatment, SBP was 126 ± 11 mmHg in the normal group and 208 ± 14 mmHg in the model and PQR groups (*P* < 0.01). However, a decrease in SBP was observed in the PQR group after 2 weeks of treatment (*P* < 0.05). After 4 weeks of treatment, the SBP of the PQR group was approximately 45 mmHg lower than at the beginning of treatment (Figure 1).

### 3.2. Morphology and Histology of Vascular Tissue Changes

**Figure 2:**
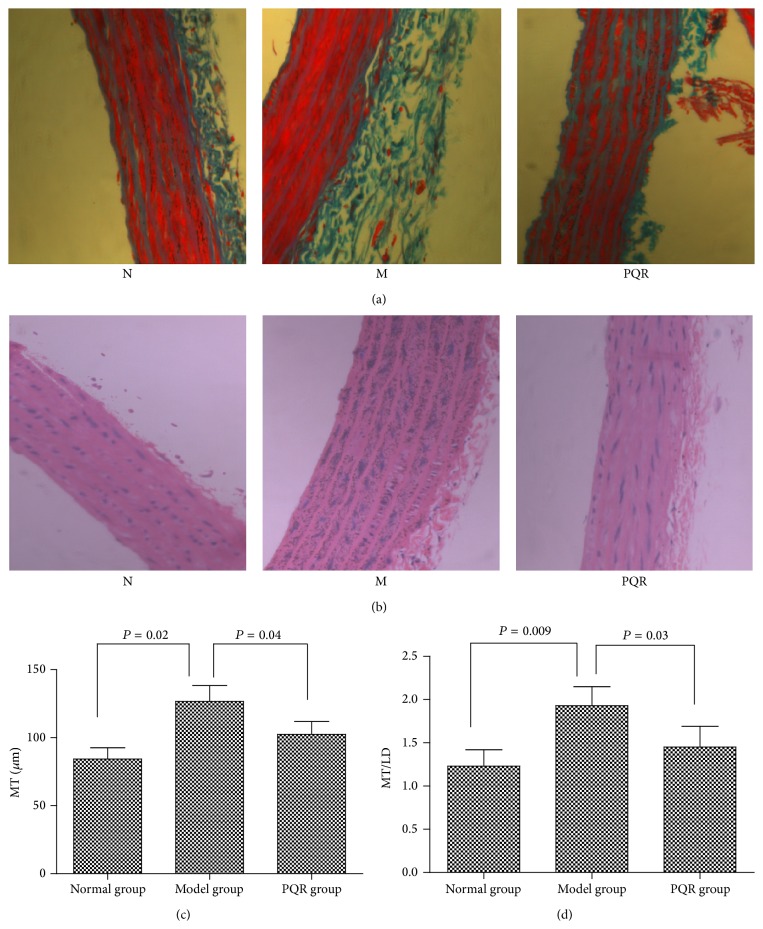
(a) Masson staining of vascular tissue in each group (400x magnification). (b) HE staining of vascular tissue in each group (400x magnification). (c) MT. (d) MT/LD. N: normal group; M: model group; PQR: PQR group. MT: medial thickness; LD: luminal diameter.

Masson and HE staining showed that the aortic tunica media of the model group was thicker than that of normal group, and the aortic tunica media of PQR-treated rats was thinner than that of control rats in the model group (Figures 2(a) and 2(b)). As shown in Figures 2(c) and 2(d), both MT and MT/LD were higher in the model group than in the normal group (MT: 126.7 ± 11.6 *μ*m versus 84.3 ± 8.3 *μ*m, resp., *P* = 0.02; MT/LD: 1.92 ± 0.19 versus 1.23 ± 0.21, resp., *P* = 0.009). However, both MT and MT/LD were significantly lower in the PQR group than in the model group (MT: 102.4 ± 9.4 *μ*m versus 126.7 ± 11.6 *μ*m, resp., *P* = 0.04; MT/LD: 1.45 ± 0.22 versus 1.92 ± 0.19, resp., *P* = 0.03).

### 3.3. Quality Assessment of Total RNA

**Figure 3:**
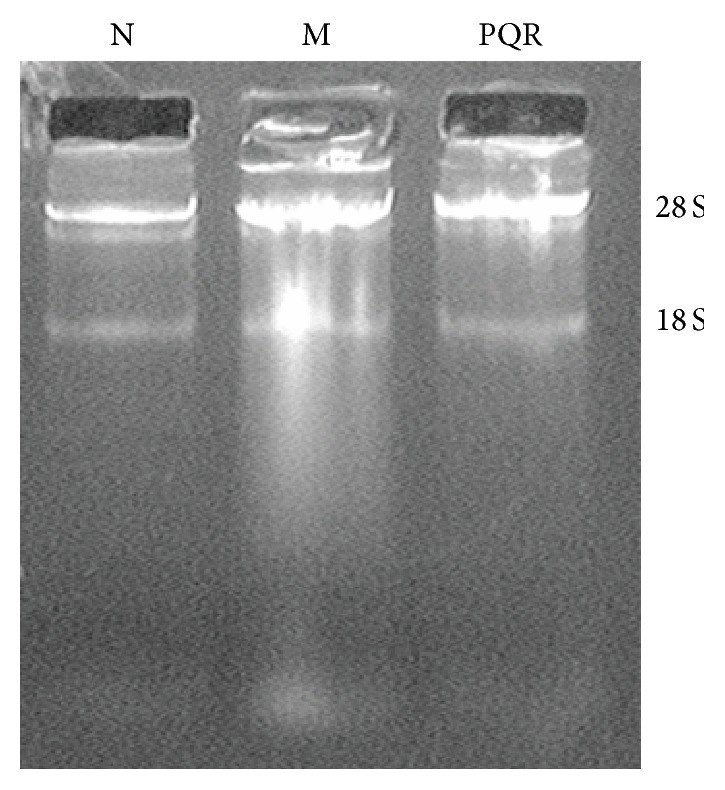
Electrophoresis of total RNA. N: normal group; M: model group; PQR: PQR group.

We extracted total RNA from the aortic tissues of all rats. The purity of the total RNA was high, as indicated by the A260/A280 ratio being greater than 1.90. Quality assessment indicated that the total RNA met the quality requirement of the miRNA microarray analysis (Figure 3 and Table 1).

### 3.4. Aberrant Expression of miRNAs in SHR Aortic Tissue

**Figure 4:**
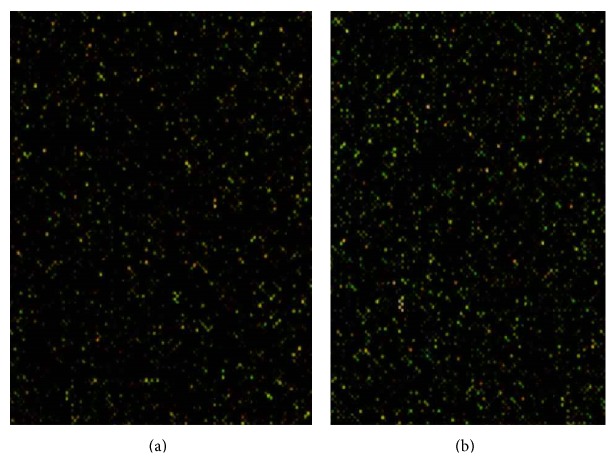
Detection of miRNA by microarray analysis. Total RNA extracted from three groups of rat aortic tissue were covalently labeled with Cy3 (green) and Cy5 (red) and hybridized to the array. The microarray slides contained two replicate subarrays. (a) Normal group and model group; (b) model group and PQR group.

To determine which miRNAs are potentially involved in the underlying mechanism of PQR treatment for essential hypertension, we tested miRNA levels in all rats by microarray analysis. We found that miRNA expression was remarkably aberrant in the model group compared with that of the normal group. In the model group, 32 of the 509 rat aortic miRNAs analyzed were differentially expressed (*P* < 0.01), with 18 miRNAs upregulated and 14 miRNAs downregulated. After 4 weeks of PQR treatment, we found that 17 of the 32 aortic miRNAs were differentially expressed; seven were upregulated and 10 were downregulated. Significant time course changes of miRNA expression were observed in the aortic tissue; more than 46.8% miRNAs were dysregulated (down- or upregulated) after PQR treatment (Figure 2(a)). All differential expression levels of miRNAs at three time points are listed in Figure 4 and Table 2. These data indicate that the development of essential hypertension involves a wave of expression of sequential classes of miRNAs. The temporal regulation of these miRNAs indicates that they might play an important role in PQR treatment of essential hypertension.

### 3.5. Validation of miRNA Microarray Results Using qRT-PCR

**Figure 5:**
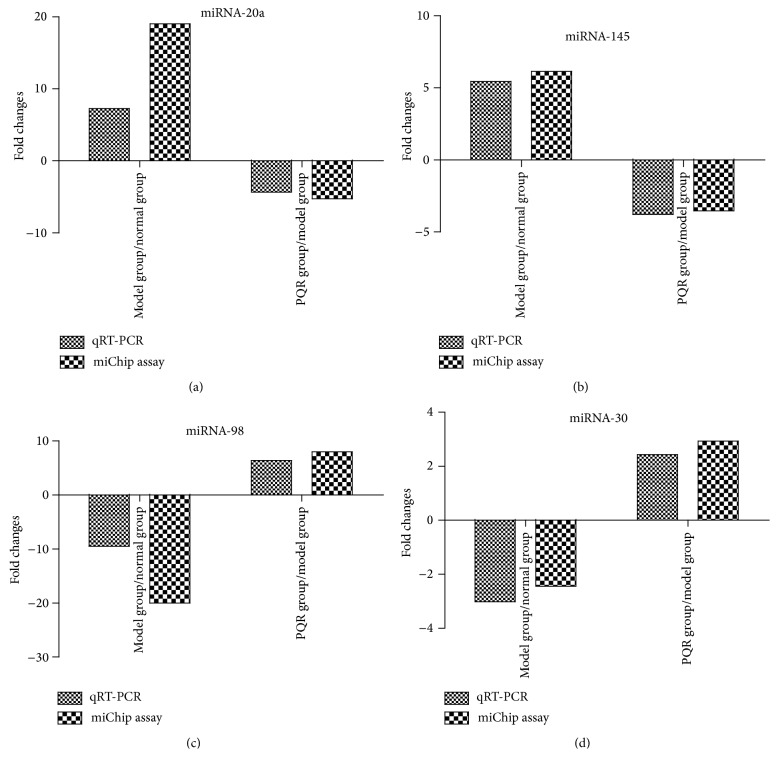
Validation of miRNA microarray data by qRT-PCR. (a)* miR-20a*; (b)* miR-145*; (c)* miRNA-98*; (d)* miR-30*. The relative expression of four miRNAs was normalized to the expression of the internal control gene (*U6*).

qRT-PCR is a quantitative and specific method that can be used to distinguish a single nucleotide difference between miRNAs. Thus, involution was obtained by miChip analysis for four selected miRNAs that showed either high (*miR-145*) or low (*miR-30*) signal intensities, or high (*miR-20a*) or low (*miRNA-98*) differential expression values among the three groups. The results of qRT-PCR analysis were often more reliable than those of the microarray analysis. qRT-PCR showed that* miR-145 *and* miR-20a *expression was downregulated in the model group compared with their expression in the PQR group, which was consistent with the microarray results. Thus, the miRNA expression profiles obtained by qRT-PCR fully support the results of miChip analysis (Figure 5).

### 3.6. Results of miR-20a Target Gene Prediction

We also performed a predicted target analysis for miRNA-20a, which was chosen because it was highly expressed in the model group and downregulated in the PQR group. Potential target genes were predicted using four software programs (miRanda, TargetScan, PicTar and DIANA-microT). To reduce false positive results, genes predicted by at least three of these four databases were selected as differentially expressed miRNA targets for subsequent analysis. Screening resulted in the selection of 38 target genes (Table 3). The target genes of miR-20a may be involved in the etiology of vascular remodeling through cell proliferation, apoptosis, migration, and differentiation.

## 4. Discussion

The observations reported here indicate that the underlying mechanism of PQR treatment for essential hypertension does not mediate vascular remodeling but strictly regulates miRNA expression. Our previous studies have shown that TCM (traditional Chinese medicine) treatment not only reduces high blood pressure in hypertension but also reverses both cardiac and vascular smooth muscle cell hypertrophy [18]. In the present study, we demonstrated that PQR treatment fully prevented the development of hypertension, as well as cardiac hypertrophy and aorta remodeling. It has been argued that excessive use of PQR in hypertension might interfere with some anatomical and/or functional parameters that are necessary to prevent blood pressure increase.

A range of evidence has demonstrated that miRNAs could be used as clinical biomarkers in essential hypertension [19]. The most robust multicenter study that provided such evidence was conducted in Ghent, Belgium, and focused on miRNA analysis of potential prognostic biomarkers in 500 neuroblastoma patients [20]. Although different technological platforms have been used for miRNA profiling, there is significant overlap between prognostic signatures described in previous work and several miRNAs that were later identified by more than three independent studies as being downregulated in essential hypertension or associated with vascular remodeling (e.g., miR-221, miR-26a, miR-21, miR-296-5p, and miR-204) [21–24].

In the present study, a microarray assay was applied to obtain miRNA expression profiles for thoracic aorta in three groups of SHR, and qRT-PCR was used to verify the microarray data. A total of 32 miRNAs in SHR (18 upregulated and 14 downregulated) and 17 miRNAs in the PQR treatment group (7 upregulated and 10 downregulated) were successfully identified. Furthermore, we also found differentially expressed miRNA-20a, with 38 potential target genes in rats, which demonstrated that miRNA expression might be significant in PQR treatment for rats with essential hypertension. In our studies, the most frequently observed and the most promising miRNAs as potential treatment targets are miR-145 [11] and miR-208 [25]. We found that miR-208 is upregulated in insulin-mediated proliferation of vascular smooth muscle cells and may promote a switch from the G0/G1 phase of the cell cycle to the S phase. The direct target of miR-208 has been shown to be p21 [25], and p21 expression in vascular smooth muscle cells has been shown to be crucial in limiting vascular proliferation in vascular remodeling, which is strongly associated with essential hypertension [26]. Interestingly, some studies [27–29] have shown that miR-143 and miR-145 play an important role in switching the phenotypes of smooth muscle cells during vascular remodeling. The function of these miRNAs is likely mediated by the degradation of many transcription factors, including KLF4, KLF5, Elk-1, and other transcription factors involved in Jagged-1/Notch signaling [30], which have been linked to the inhibition of differentiation of smooth muscle cells. MiR-20a, a member of the miR-17–92 cluster, is a highly conserved miRNA within a noncoding RNA encoded by the c13 or f25 host gene localized on chromosome 13 [31]. The functions of each cluster member in essential hypertension have not been clearly established. Recently, Pin et al. found that miR-20a can inhibit the expression of MKK3 and downregulate p38 pathway-mediated and VEGF-induced endothelial cell migration and angiogenesis [32]. miR-20a has also been shown to play an important role in vascular remodeling [33]. In contrast, several functionally well-characterized miRNAs that had previously been observed in other diseases were later identified in SHR for the first time with a high level of statistical significance, indicating their potential involvement in essential hypertension pathogenesis. These included miR-20a, miR-18b, miR-375, and miR-215 [34].

In conclusion, our study demonstrates that PQR has beneficial effects in reducing blood pressure and vascular remodeling in SHR. The underlying mechanism might be related to the modulation of 18 upregulated and 14 downregulated miRNAs, in particular, miR-20a, miR-145, miR-30, and miR-98. We suggest that the target genes of miR-20a may be involved in the etiology of vascular remodeling through cell proliferation, apoptosis, migration, and differentiation. However, the underlying mechanisms should be further investigated through basic research and well-controlled clinical trials.

## 5. Conclusion

Taken together, our findings indicated that PQR could exert its antihypertensive effect through deterioration of the vascular remodeling process. The mechanism might be associated with regulating differentially expressed miRNAs in aorta tissue.

## Figures and Tables

**Table 1:** A260, A280, and A260/A280 ratios, and miRNA concentrations.

Group	A260	A280	Ratio of A260/A280	Concentration (*µ*g/uL)
Normal group	0.57	0.28	1.96	0.183
Model group	1.06	0.51	2.08	0.295
PQR group	0.92	0.47	1.95	0.266

**Table 2:** Significantly upregulated and downregulated miRNAs in three groups.

miRNA	Expression level			Model/normal	PQR/model
	Normal group	Model group	PQR group		
rno-miRNA-1	36.3	82.4	68.7	2.27	0.83
rno-miRNA-10a/b	8.5	21.2	11.8	2.49	0.56
rno-miRNA-17-5p	12.1	93.3	28.9	7.71	0.31
rno-miRNA-20a	32.7	621.6	121.5	19.01	0.19
rno-miRNA-96	43.2	753.7	211.3	17.45	0.29
rno-miRNA-126-5p	9.3	32.3	35.6	3.47	1.10
rno-miRNA-139	19.7	42.8	33.4	2.17	0.78
rno-miRNA-145	12.8	78.6	23.5	6.14	0.30
rno-miRNA-153	6.8	105.9	35.1	15.57	0.33
rno-miRNA-186a	35.5	213.6	178.8	6.52	0.84
rno-miRNA-187	26.4	136.6	33.4	5.17	0.24
rno-miRNA-196a/b	45.1	209.7	61.2	4.65	0.29
rno-miRNA-210	25.3	198.8	38.6	7.86	0.19
rno-miRNA-218	19.4	79.3	54.8	4.09	0.61
rno-miRNA-221	22.5	89.5	29.8	3.98	0.33
rno-miRNA-378	14.8	125.3	38.7	8.47	0.31
rno-miRNA-451	34.5	76.4	59.8	2.21	0.78
rno-miRNA-486	7.1	23.5	22.8	3.31	0.97
rno-miRNA-556	12.4	61.7	23.5	4.97	0.38
rno-miRNA-15b	164.3	23.8	28.9	0.14	1.21
rno-miRNA-26a/b	87.4	15.6	47.9	0.18	3.13
rno-miRNA-30	79.5	32.3	94.8	0.41	2.93
rno-miRNA-23a/b	23.5	6.8	5.7	0.29	0.84
rno-miRNA-29b	256.2	45.9	138.2	0.18	3.01
rno-miRNA-98	135.1	6.6	52.7	0.05	7.98
rno-miRNA-122	120.6	19.7	78.6	0.16	3.99
rno-miRNA-125b	378.6	113.4	178.2	0.29	1.57
rno-miRNA-142-3p	99.6	48.7	46.9	0.49	0.96
rno-miRNA-158	132.8	29.8	34.2	0.22	1.15
rno-miRNA-21	56.6	10.3	142.7	0.18	13.85
rno-miRNA-330	322.5	80.9	118.6	0.25	1.47
rno-let-7b/c	78.6	17.4	15.2	0.22	0.87

**Table 3:** Predicted target genes of miRNA-20a.

Target gene	Accession no.	Target gene name
ZNFX1	NM_021035	Zinc finger, NFX1-type containing 1
IL25	NM_022789	Interleukin 25
MAP3K2	NM_006609	Mitogen-activated protein kinase kinase kinase 2
AMPD3	NM_001025390	Adenosine monophosphate deaminase 3
GPR137C	NM_001099652	G protein-coupled receptor 137C
ACTBL2	NM_001017992	Actin, beta-like 2
MFAP3L	NM_001009554	Microfibrillar-associated protein 3-like
TRIP11	NM_004239	Thyroid hormone receptor interactor 11
DGUOK	NM_080918	Deoxyguanosine kinase
MFN2	NM_001127660	Mitofusin 2
VPS36	NM_004755	Vacuolar protein sorting 36 homolog
PLS1	NM_001145319	Plastin 1
ARHGAP12	NM_018287	Rho GTPase activating protein 12
FZD3	NM_017412	Fizzled family receptor3
PDK4	NM_002612	Pyruvate dehydrogenase kinase, isozyme 4
KIF23	NM_004856	Kinesin family member 23
VLDLR	NM_003383	Very low density lipoprotein receptor
FBXO4B	NM_001024680	F-box protein 4B
ZNF652	NM_014897	Zinc finger protein 652
RASD1	NM_016048	RAS, dexamethasone-induced 1
RS1	NM_000330	Retinoschisin 1
TNFRSF21	NM_014452	Tumor necrosis factor receptor superfamily, member 21
FGL1	NM_004467	Fibrinogen-like 1
CCND2	NM_001759	Cyclin D2
TMEM133	NM_032021	Transmembrane protein 133
LPGAT1	NM_014873	Lysophosphatidylglycerol acyltransferase 1
IPO7	NM_006391	Importin 7
GUCY1A3	NM_000856	Guanylate cycle 1, souble, alpha 3
TSPAN9	NM_001168320	Tetraspanin 9
KLF12	NM_007249	Kruppel-like factor 12
SMOC2	NM_001166412	SPARC related modular calcium binding 2
MAP3K3	NM_002401	Mitogen-activated protein kinase kinase kinase 3
NRP2	NM_018534	Neuropilin 2
SOCS6	NM_004232	Suppressor of cytokine signaling 6
SLC16A6	NM_001174166	Solute carrier family 16, member 6 (monocarboxylic acid transporter 7)
PRR14L	NM_173566	Proline rich 14-like
ANO6	NM_001025356	Anoctamin 6
ZBTB43	NM_001135776	Zinc finger and BTB domain containing 43
